# Harbin consensus conference and quality of infertility trials: reflections of a scientist on the Italian experience

**DOI:** 10.1186/1757-2215-6-81

**Published:** 2013-11-20

**Authors:** Stefano Palomba

**Affiliations:** 1 Obstetrics and Gynecology Unit, Department of Obstetrics, Gynecology and Pediatrics, Azienda Ospedaliera “Santa Maria Nuova”, Istituto di Ricovero e Cura a Carattere Scientifico, University of Modena and Reggio Emilia, Via Risorgimento 80, Reggio Emilia 42123, Italy

**Keywords:** CONSORT, Infertility, RCT, Reproductive medicine, Sterility

## Abstract

During the days August 22–24, 2013 has been held in Harbin (China) an International Consensus Conference aimed to improve the quality and the reporting of the randomized controlled trials (RCTs) in infertility and subfertility field. I, as Italian scientist with experience in clinical infertility trials, was invited to have a speech on the Italian experience in RCTs, with particular regard for the surgical trials. Considerations on this subject were particularly interesting to highlight pitfalls and triumphs of research in Italy.

## 

Italy, an old Country in the heart of the old Continent. Many know Italy for good food, the friendliness of its people, its natural beauty and its history. Today, Italy is one of the main European Countries and industrialized Nations. Italy is also considered a scientifically advanced state, with particular attention to the legislation in the field of scientific research [1], and Italians are often known as smart, genial and intelligent people. Furthermore, the results of scientific research in Italy are less bright. In fact, Italian journals published fewer articles annually and fewer RCTs, have a low citation, a low *Hirsh* factor and low impact factors (IF) as demonstrated in a recent study aimed to investigate a possible relationship between editorial leadership and journal quality in Italy and United Kingdom (UK) [2]. In addition, only a little proportion of Italian journals require statements about funding, conflict of interest and registration of clinical trial and none of them adheres to international guidelines, such as the Committee on Publication Ethics (COPE), the CONsolidated Standards Of Reporting Trials (CONSORT) or the Quality Of Reporting of Meta-analysis (QUORUM) [2]. That figure seems to be due to the underfunded research [3,4] and low use of meritrocracy, leading to “brain drain” phenomenon [5,6].

## Search strategy

In order to assess the quantity and quality of the Italian RCTs, and their transparency, I performed a systematic review including two-arm parallel RCTs on human infertility performed in Italy in the 18 years from 1996 to July 2013. The lower limit for the research was defined considering the publication year of the first paper on CONSORT guidelines [7]. Papers with both English and Italian language were included in order to not lose RCTs in original language, and the main electronic databases, websites of the electronic registers for Clinical trials, and of the main Italian Scientific Societies of Reproductive Medicine were checked. Only non-Italian papers (according to the affiliation of first author), semi-randomized or cross-over studies, and all papers published as abstract form were excluded. General terms as infertility, sterility, and reproduction were matched with several specific terms including diseases, as polycystic ovary syndrome (PCOS), uterine fibroids, endometriosis, and interventions, both pharmacological, as gonadotrophins, clomiphene, metformin, and non-pharmacological, as laparoscopy or surgery.

After the papers’ selection, general information (including year of publication, journal of publication, collaboration with different countries) and specific information (including all 37 items/sub-items of the last revision of the CONSORT guidelines) [8] were noted. Specifically, each item was evaluated for all included papers, also for those published before 2010, and a score of 1 was given in case of clear information and of 0 for unavailable or unclear information. All data were extrapolated by an Italian Clinical Research Organization (FullCRO of Rome, Italy) expert in medical writing from manual examination, and checked by me. In addition, papers that followed formally CONSORT guidelines, the funding source (classified as governmental agencies, private not for profit organizations, industry funding, explicit statement of no funding, or funding source not reported)/conflict of interest, the excellence in the study design (superiority, noninferiority, equivalence), the type of result (positive or negative according to *P* value), type of intervention (therapeutic or diagnostic, and surgical or non-surgical), type of controls were also noted.

## General data

After selection, a total of 111 papers were identified and included in the final analysis.

The first finding that emerged was a proportion of about 5% in two-arm parallel RCTs (considering an overall amount of RCTs of 2,225). In more than an half of cases the papers regarded pharmacological intervention, whereas in 19% and 24% of cases surgical trials and use of supplements or biological mechanisms, respectively. In about two third of cases the field of interest was the gynecology, whereas in 14% was the andrology or the reproductive biology.

In about 70% of cases, papers were published on *Fertility and Sterility* and *Human Reproduction* with a slight prevalence of papers published on *Fertility and Sterility*. Only few papers were published on journals with higher IF, as the *Journal of Clinical Endocrinology and Metabolism* (JCEM).

**Figure 1:**
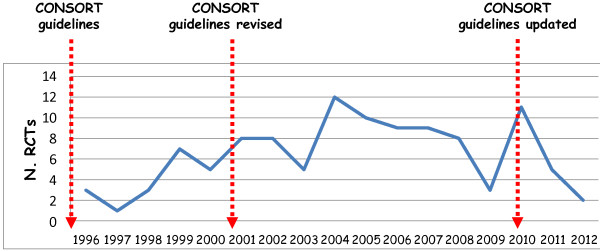
Number of Italian RCTs per year.

The evaluation of the number of published RCTs per year showed a trend of increase during the first years just after the 2001 and a rate essentially stable over the last 10 years with no effect of the CONSORT guidelines publication [7-9] (Figure 1).

**Figure 2:**
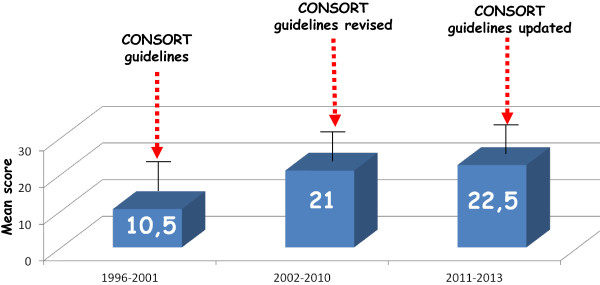
Overall CONSORT score for three time intervals.

Considering the CONSORT items and the potential total score ranging from 0 to 37, a suboptimal total score (in median) for Italian papers of 24 (range from 4 to 30) was obtained. Moreover, the distribution of the median scores for publication year showed a significant increase over the years. That increase was even more evident after grouping the papers for three time intervals defined according to publication year of the CONSORT guidelines and their two revisions (Figure 2).

**Figure 3:**
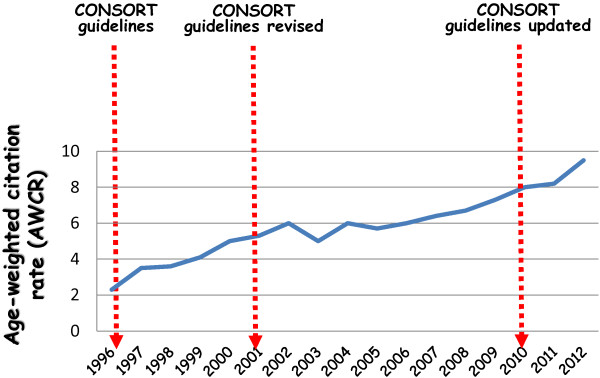
Age-weighted citation rate (AWCR) per year.

Using the SCOPUS program and the age-weighted citation rate (AWCR) as bibliometric parameter, a constant increase over the time for the Italian RCTs was observed in papers quality (Figure 3). Then, groping of the Italian papers in two categories, i.e. low-scored papers (overall CONSORT score lower than 24) and high-scored papers (overall CONSORT score equal or higher than 24), a difference between two groups in mean AWCR of at least 10-fold; this different was even more evident considering the median AWCR. Finally, a regression analysis demonstrated a direct relationship between AWCR and CONSORT score (*r* = 0.645, *P* = 0.020) and how the clarity in scientific writing increases closely the citations rate.

Moreover, data on the relationship between transparency in scientific writing and citations rate require some thought. All authors should know that a clearly written paper is more likely to be cited and this, simply, for technical aspects. For example, a manuscript with clear title, inclusion/exclusion criteria and results permit its inclusion in systematic reviews or meta-analyses, or a detailed methodology enhances the quotation of diagnostic and/or therapeutic techniques in materials and methods of further papers. However, the risk is to confound the “scientific transparency” with the “scientific quality”. In fact, the risk is the use of the CONSORT guidelines as editorial tool to assess the acceptability of a manuscript or, conversely, to improve its transparency for improving the journal citation index and impact factor. In this case we’ll have papers masked by “good trials” with an high citation index, independently from their true scientific quality. In other words, the Editors should not use CONSORT guidelines to accept or reject manuscripts, even if it is their ethical obligation to support peer reviewers to strive for transparent and accurate reporting of research [10].

## Data analyzed according to the item/sub-item of the CONSORT checklist

In arbitrary classification of the transparency of the Italian papers was given for each item/sub-item of the CONSORT checklist [critical (lower than 25%), poor (range 26-50%), sub-optimal (51-75%), and optimal (>75%) transparency] in order to define the critical areas to improve.

Considering the first item of the CONSORT checklist, including the identification as a randomized trial in the title and the writing of a structured summary, the total scores were 66.7 and 61.1%, respectively (Additional file 1: Table S1). That result can be considered apparently good. However, the evaluation of the extension of CONSORT guidelines to abstract [11] and to non-pharmacological trials [12], revealed an adherence to items/sub-items very low with a proportion of papers reduced to a critical 0% if all items/sub-item specified are considered in the analysis. The reason for this figure could be due to the words count limitation for abstract writing, issue particularly important for non-anglophone countries, to the publication of RCTs as correspondence (see *Fertility & Sterility*) or brief report (see *JCEM*), and to conflicting Authors’ guidelines between suggestions for CONSORT guidelines and for (in-)appropriate abstracts formats. Fortunately, in the last years, the Web is offering increasing opportunities to address these problems and many journals offer the possibility to put additional material on the Web only without words limit [13].

The scores obtained by Italian papers regarding the evaluation of the Introduction section, including the items on the scientific background and explanation of rationale and the specific objectives or hypotheses, were very high with proportions that can be considered optimal (Additional file 1: Table S1). On the other hand, the figure is extremely variable when we consider the items employed for the Methods. In general, I identified some critical areas regarding the changes in trial design and in trial outcome after trial start, the personnel who generated the allocation sequence and assigned participants to interventions, and the statistical methods for additional analysis and sub-analyses.

However, more interesting data for non-Italian researchers are related probably to other specific aspects. Firstly, it is very unclear in the Italian papers the relationship between affiliation, setting and locations. In 68% of cases, the paper was written under multiple affiliations, but if we analyze the real proportion of multicenter studies it was of 6% alone. This mean that, notwithstanding Italy is a small country, patients are enrolled and treated essentially in only one center limiting the external validity of the findings. In addition, the studies were conducted in collaboration with other countries in only the 3% of cases. This point is particularly important for Infertility research considering that in Italy the pharmacological experimentation is regulated by rigorous legislation and vigilated by Italian Medicines Agency (AIFA), as only 114 of the 351 public infertility centers could perform phase III and phase IV research and several private infertility centers with extensive experience in infertility management cannot carry out the majority of clinical trials unless they collaborate with public centers. Thus, in Italian papers it should be crucial to specify the mean of multiple affiliations and to clarify the “true” setting. On the other hand, in Italy there is no specific and clear regulation regarding the surgical experimentations and small private centers with a low volume and surgeons without specific certifications can perform clinical research.

The second point is that none of the included RCTs was “pragmatic”, term initially coined to define a trial designed to help one to choose between options for care [14]. To date, a “pragmatic” trial can be defined as a reality-based RCT aimed to change the clinical practice. Several explanations may be invoked: economic (as the reduction of funds for the research), organizational (as the lack of a coordination from main Italian Scientific Societies), and cultural (design of RCTs in little well selected patients’ populations). As consequence, one witnesses many biological or explanatory RCTs in Italy which have optimal internal validity but a low reproducibility or external validity [15] since strict eligibility criteria can make the study sample atypical, unrepresentative, and irrelevant from a clinical point of view [16]. Moreover, the use of well selected patients’ samples is a tendency very common in European Countries. In fact, one of the concepts very dear to European researchers is the “therapeutic tailoring”, concept not specific for the reproductive medicine, but also applied to other scientific areas such as gynecological oncology and/or postmenopausal hormone replacement therapy. This figure seems similarly to reflect the trend toward the tailor made typical of the Italian sartoria.

Another interesting issue is the definition of the interventions in the Italian papers. In my analysis, a clear and detailed description of the intervention was provided in a high proportion of papers, but if we analyze the interventions considered as “control” they were not “standard care”. In fact, in Italy national guidelines for the “good clinical practice” in infertility are not available and none of the many Italian scientific societies drafted clinical guidelines. In addition, the Law 40 for the assisted reproductive technologies has also been overtaken by subsequent judgments of the Constitutional Court.

The blinding procedure in Italian papers was also adequately reported in a high proportion of cases, although the rate of blinding studies was very low (double-blinding, single-blinding, assessor-blinding in 6%, 7%, and 16% of the studies, respectively). However, the blinding procedure is not a primary indicator of overall quality of the trial [17-19] especially for the infertility trials where the primary outcome is or should be dichotomous [20,21].

In the evaluation of the CONSORT items employed for the methods, an extremely variable picture was again observed (Additional file 1: Table S1). Critical areas related to the data analysis, i.e. use of intention-to-treat (ITT) principle and expression as absolute and relative effect size, the ancillary analyses and the harms.

Although clarifications about the participants’ flow chart and the baseline characteristics are given in a high proportion of papers (Additional file 1: Table S1), careful evaluation of all studies showed that only a small percentage of the studies reported to have followed the ITT principle had indeed performed a “true” ITT analysis [22]. Similarly, the evaluation of papers not reporting any specific data analysis demonstrated a right use of the per protocol (PP) analysis in about 14% alone. In fact, several reasons were considered “arbitrary” criteria to exclude a patients when it is used both ITT and PP analysis. Of particular interest was the exclusion of patients who obtained a pregnancy after the randomization and before the treatment start; in these cases the authors considered the outcome non-related to the intervention and, thus erroneously excluded the patients from final analysis. Un-intentional events due to ITT in good prognosis patients have been also reported frequently in international literature [23-25]. Conversely, in poor prognosis patients, a long post-randomization time-to-intervention could result in a change in the baseline patients’ characteristics (age-related ovarian response in aged patients). Thus, it is clear that the intervention in the infertility trials should start just after randomization and long time intervals between randomization and start of treatment should be avoided.

Another critical point relates to ancillary analyses. Ancillary analyses can be useful in terms of therapeutic tailoring, evaluation of a hypothesis or of a mechanism of action to confirm a biological plausibility, but are also related to several concerns including false positive findings for underpowered data (and consequent type 2 error), production of data with poor external validity and, especially in the industry-funded trials, spining for “positive result”. However, when ancillary analyses are detailed in the papers, in 22% and 17% of cases they were exploratory and pre-specified, respectively, and in about 60% of cases their aim was unclear. Of interest, in no case the industry funded the study as contrarily reported by international experience [26] and, also of interest, the inverse relationship observed between results from primary outcome and results of ancillary analyses. In particular, in case of RCTs with positive results of the primary outcome, the proportion of subanalyses with negative results was higher, whereas in case of RCTs with negative results of the primary outcome, the proportion of subanalyses with positive results was higher.

In less than 30% of the Italian papers assessed and detailed the harms, the major mistakes in reporting harms-related data [27] were the use of generic or vague statements, the use of cumulative numbers for all adverse events failing to provide data for the type, severity, the timing of events, the lack of data on patients with one or multiple adverse events, the lack of safety data according to ITT analysis.

The explanation for this figure can be, as suggested by Legro [28], that “…The safety hypothesis is (too much times) implicit in any primary efficacy hypothesis..”. However, the risk data is more complex than efficacy data since they are not always dichotomous and should be assessed also after long-term follow-up and intervention termination. These are the cases of the assessment of long-term health of babies born from new technologies or the maternal treatment-related cancer risk.

Several suggestions have been provided in the extension CONSORT guidelines to non-pharmacological trials that include surgical RCTs [12]. The concerns for surgical trials in infertility relate essentially the selection of the centers and of the surgeons, the standardization of each procedure (including instrumentation and team) that should be detailed step-by-step considering and standardizing also potential co-interventions [12]. In addition, all data on the surgeons, the procedures really performed for each arm and the Centers where they have been performed should be reported as results. In this regard and considering the items of the extension CONSORT guideline to non-pharmacological trials [12], none of the Italian trials satisfied them.

The data obtained for the items of the discussion section were generally good. However, although only a little proportion of RCTs reported totally innovative interventions (~6%), in none of the discussion in the Italian RCTs included systematic reviews or meta-analyses. It is possible however that the best way to discuss the findings is to include them in the context of the previous systematic review with updating of the data synthesis [29].

Considering the last three CONSORT items named “other information”, the transparency of the Italian papers is to be considered “critical”. Only a very low proportion of RCTs were registered, had a protocol available for consultation, and reported the funding source. Specifically, notwithstanding the lack of public funds for the research in Italy, only 4% of included RCTs were industry-funded. The careful evaluation of the Italian papers demonstrated no conflict of interest in a proportion of about 90% of cases. However, in 8 cases (7.3%) a person of pharmaceutical company was included as co-Authors. Surprisingly, also in these cases the funding source was not reported and no conflict of interest was declared.

However, that results show the interest of pharmaceutical companies in the clinical research that, if transparent and well-regulated, could be a factor well received, especially when the public funding for research are strongly reduced.

## Conclusion

Notwithstanding the several limitations of my analysis, it identifies the “weight” of the Italian scientific research and the “typology” of the Italian clinical studies, essentially single-center, explanatory, and on well selected populations. Although there is no specific and formal training in research methods in Italy, the quality of Italian research in infertility is constantly increasing. Further meetings like the one held in Harbin are welcome. These may be useful to give a survey of the situation nationally and internationally in the field of Reproductive Medicine, and in the constructive spirit to propose new strategies to improve the quality and the transparency of the research.

## Supplementary Material

Additional file 1: Table S1 Item-by-item analysis of adherence to the CONSORT 2010 in Italian infertility RCTs. [11]. Click here for file
