# Supplementary material for: Harbin consensus conference and quality of infertility trials: reflections of a scientist on the Italian experience
Source: J Ovarian Res. 2013 Nov 20;6:81. doi: 10.1186/1757-2215-6-81 (PMC3843534; doi:10.1186/1757-2215-6-81)
Supplement: Additional file 1: Table S1 — Item-by-item analysis of adherence to the CONSORT 2010 in Italian infertility RCTs. [11]. [file 1757-2215-6-81-S1.doc]

Table S1. Item-by-item analysis of adherence to the CONSORT 2010 in Italian infertility RCTs.

| Section/Topic | Item No | Checklist item | Percentage of adherence |
| --- | --- | --- | --- |
| Title and abstract | | | |
|  | 1a | Identification as a randomised trial in the title | 66.6% |
|  | 1b | Structured summary of trial design, methods, results, and conclusions (for specific guidance see CONSORT for abstracts) | 61.1% - 0%* |
| Introduction | | | |
| Background and objectives | 2a | Scientific background and explanation of rationale | 94.4% |
|  | 2b | Specific objectives or hypotheses | 88.9% |
| Methods | | | |
| Trial design | 3a | Description of trial design (such as parallel, factorial) including allocation ratio | 82.9% |
|  | 3b | Important changes to methods after trial commencement (such as eligibility criteria), with reasons | 16.2% |
| Participants | 4a | Eligibility criteria for participants | 100% |
|  | 4b | Settings and locations where the data were collected | 31.5% |
| Interventions | 5 | The interventions for each group with sufficient details to allow replication, including how and when they were actually administered | 77.5% |
| Outcomes | 6a | Completely defined pre-specified primary and secondary outcome measures, including how and when they were assessed | 62.2% |
|  | 6b | Any changes to trial outcomes after the trial commenced, with reasons | 23.4% |
| Sample size | 7a | How sample size was determined | 63.1% |
|  | 7b | When applicable, explanation of any interim analyses and stopping guidelines | 27.9% |
| Randomisation: |  |  |  |
| Sequence generation | 8a | Method used to generate the random allocation sequence | 57.7% |
|  | 8b | Type of randomisation; details of any restriction (such as blocking and block size) | 36.0% |
| Allocation concealment mechanism | 9 | Mechanism used to implement the random allocation sequence (such as sequentially numbered containers), describing any steps taken to conceal the sequence until interventions were assigned | 35.1% |
| Implementation | 10 | Who generated the random allocation sequence, who enrolled participants, and who assigned participants to interventions | 18.9% |
| Blinding | 11a | If done, who was blinded after assignment to interventions (for example, participants, care providers, those assessing outcomes) and how | 58.6% |
|  | 11b | If relevant, description of the similarity of interventions | 34.2% |
| Statistical methods | 12a | Statistical methods used to compare groups for primary and secondary outcomes | 90.1% |
|  | 12b | Methods for additional analyses, such as subgroup analyses and adjusted analyses | 21.6% |
| Results | | | |
| Participant flow (a diagram is strongly recommended) | 13a | For each group, the numbers of participants who were randomly assigned, received intended treatment, and were analysed for the primary outcome | 79.3% |
|  | 13b | For each group, losses and exclusions after randomisation, together with reasons | 73.9% |
| Recruitment | 14a | Dates defining the periods of recruitment and follow-up | 75.7% |
|  | 14b | Why the trial ended or was stopped | 100% |
| Baseline data | 15 | A table showing baseline demographic and clinical characteristics for each group | 86.5% |
| Numbers analysed | 16 | For each group, number of participants (denominator) included in each analysis and whether the analysis was by original assigned groups | 36.0% |
| Outcomes and estimation | 17a | For each primary and secondary outcome, results for each group, and the estimated effect size and its precision (such as 95% confidence interval) | 34.2% |
|  | 17b | For binary outcomes, presentation of both absolute and relative effect sizes is recommended | 5.4% |
| Ancillary analyses | 18 | Results of any other analyses performed, including subgroup analyses and adjusted analyses, distinguishing pre-specified from exploratory | 17.5% |
| Harms | 19 | All important harms or unintended effects in each group (for specific guidance see CONSORT for harms) | 27.9% |
|  |  | Using generic or vague statements | 100% |
|  |  | Failing to provide separate data for each study arm | 51.6% |
|  |  | Providing summed numbers for all adverse events for each study arm, regardless of type, severity or seriousness | 76.4% |
|  |  | Reporting only harms with high frequency | 25.8% |
|  |  | Reporting only harms that reach a P value threshold between arms | 29.0% |
|  |  | Reporting measures of central tendency without extreme values | 19.4% |
|  |  | Improperly report the timing of the events | 87.1% |
|  |  | Not distinguishing between patients with one and multiple adverse events | 87.1% |
|  |  | Not providing statistical analysis for events | 48.4% |
|  |  | Not providing data on harms for all randomly assigned participants | 83.9% |
| Discussion | | | |
| Limitations | 20 | Trial limitations, addressing sources of potential bias, imprecision, and, if relevant, multiplicity of analyses | 43.2% |
| Generalisability | 21 | Generalisability (external validity, applicability) of the trial findings | 63.1% |
| Interpretation | 22 | Interpretation consistent with results, balancing benefits and harms, and considering other relevant evidence | 72.1% |
| Other information | | |  |
| Registration | 23 | Registration number and name of trial registry | 7.2% |
| Protocol | 24 | Where the full trial protocol can be accessed, if available | 2.7% |
| Funding | 25 | Sources of funding and other support (such as supply of drugs), role of funders | 3.6% |

*Abstract extension (Hopewell et al., 2008).
